# Recent Advances in Raman Spectral Classification with Machine Learning

**DOI:** 10.3390/s26010341

**Published:** 2026-01-05

**Authors:** Yonghao Liu, Yizhan Wu, Junjie Wang, Jiantao Qi, Changjing Zhou, Yuhua Xue

**Affiliations:** 1 College of New Energy, China University of Petroleum (East China), Qingdao 266580, China; s24150029@s.upc.edu.cn (Y.L.); 2215060114@s.upc.edu.cn (Y.W.); zhouchangjing78@163.com (C.Z.); 2 School of Petroleum Engineering, China University of Petroleum (East China), Qingdao 266580, China; 2202030217@s.upc.edu.cn; 3 State Key Laboratory of Marine Coatings, Ocean Chemical Industry Research Institute Co., Ltd., Qingdao 266071, China; xueyuhua163@163.com

**Keywords:** Raman spectroscopy, machine learning, deep learning, spectral classification

## Abstract

Raman spectroscopy is a non-destructive analytical technique based on molecular vibrational properties. However, its practical application is often challenged by weak scattering signals, complex spectra, and the high-dimensional nature of the data, which complicates accurate interpretation. Traditional chemometric methods are limited in handling complex, nonlinear Raman data and rely on tedious, expert-knowledge-based feature engineering. The fusion of data-driven Machine Learning (ML) and Deep Learning (DL) methods offers a robust solution, enabling the automatic learning of complex features from raw data and achieving high-accuracy classification and prediction. The present study employed a structured narrative review methodology to capture the research progress, current trends, and future directions in the field of ML-assisted Raman spectral classification. This review provides a comprehensive overview of the application of traditional ML models and advanced DL architectures in Raman spectral analysis. It highlights the latest applications of this technology across several key domains, including biomedical diagnostics, food safety and authentication, mineralogical classification, and plastic and microplastic identification. Despite recent progress, several challenges remain: limited training data, weak cross-dataset generalization, poor reproducibility, and limited interpretability of deep models. We also outline practical directions for future research.

## 1. Introduction

Raman spectroscopy is a non-destructive, label-free molecular vibrational spectroscopic technique with applications across numerous fields, including materials science, biomedicine, food safety, and environmental monitoring [1,2,3]. The technique is based on the inelastic scattering of photons from a substance, where analysis of the frequency shifts in the scattered light provides a unique molecular “fingerprint” [4]. Raman spectroscopy offers high molecular specificity and requires minimal sample preparation. However, weak scattering signals, complex spectra, instrument drift, and environmental interference often hinder reliable interpretation [5,6]. Raman spectra are reported as intensity versus Raman shift (cm^−1^), where peaks reflect molecular vibrational modes. In practical measurements, fluorescence background, cosmic-ray spikes, and intensity variation are common and are usually handled by baseline correction/despiking and intensity normalization before multivariate analysis. In this review, SERS refers to surface-enhanced Raman spectroscopy, while CARS/SRS denotes coherent Raman modalities that improve sensitivity or speed.

Traditionally, Raman spectral data analysis has relied on chemometric methods for feature extraction and dimensionality reduction to enable classification or quantitative analysis. However, these methods are often based on the assumption of data linearity, which presents a significant limitation when dealing with the complex, high-dimensional, and nonlinear characteristics of Raman data. This approach also requires extensive feature engineering based on expert knowledge, which is often tedious and inefficient [7,8]. With the advent of AI, data-driven machine learning (ML) methods are increasingly being integrated with Raman spectroscopy. Unlike traditional approaches that struggle with complex, high-dimensional data, ML can automatically learn features directly from raw data and perform accurate classification and prediction [9]. Models such as Support Vector Machines (SVMs), Random Forests (RFs), and neural networks have shown excellent performance in Raman spectral classification. They have been successfully applied in fields like material identification, biomedical diagnostics, and food quality analysis, demonstrating the potential for rapid, high-throughput, and automated analysis [10,11,12]. From an ML perspective, Raman spectra are shaped not only by chemical composition but also by measurement conditions. Spectrometer and acquisition settings (such as excitation wavelength, laser power, integration time, spectral resolution, and optical geometry) affect SNR, fluorescence background, intensity variability, and spectral drift [13]. These parameters should be recorded as standardized metadata to support reproducibility and cross-instrument generalization. A preprocessing workflow includes despiking (cosmic-ray removal), wavenumber/intensity calibration, baseline correction, and normalization [14]. Since preprocessing can change the learned representation, parameters and code should be reported to enable fair benchmarking.

Despite rapid advances in Raman–ML research, existing reviews often emphasize either deep learning (DL) architectures or a single application domain, and cross-study comparison is frequently hindered by inconsistent reporting of dataset size, preprocessing, and validation design. Therefore, this review provides a workflow-oriented synthesis across multiple application domains. We relate measurement and preprocessing choices to model robustness, compare representative ML/DL methods using consistent reporting of datasets and validation, and summarize practices that improve reproducibility and deployment.

## 2. Scope and Review Methodology

This article presents a structured narrative review of the recent progress in ML-based Raman spectral classification. It is not intended to be a systematic review with a formal, pre-registered protocol. Instead, we provide a representative overview of key trends, applications, and challenges in ML-assisted Raman classification, and we summarize practical guidance for method selection and reporting. The literature was surveyed from major academic databases, including the Web of Science and Google Scholar, covering a period primarily from 2020 to 2025, to focus on the most current advancements. Inclusion criteria prioritized peer-reviewed articles that demonstrated a clear integration of a specific ML/DL model with Raman spectroscopy for a classification task and reported clear performance metrics. We focused on studies that were illustrative of significant trends, such as the use of novel deep learning architectures, applications in emerging fields, or innovative approaches to data handling and model interpretability.

**Figure 1:**
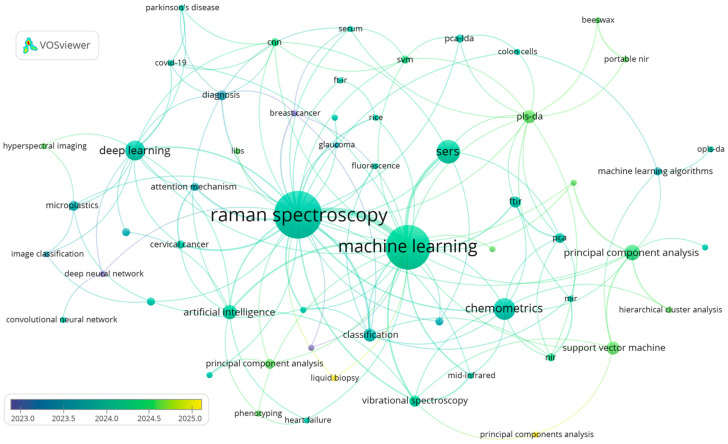
Keyword co-occurrence network for ML–Raman spectroscopy research from 2023 to 2025.

Figure 1 presents a keyword co-occurrence network reflecting research hotspots in the integration of ML with Raman spectroscopy from 2023 to 2025. The map was generated using VOSviewer (version 1.6.20) based on records retrieved from the Web of Science Core Collection, following standardized bibliometric workflows widely adopted for trend analysis and thematic mapping. In this network, node size reflects keyword frequency, while link strength represents co-occurrence relationships, allowing the identification of both dominant topics and emerging connections. Core terms such as “Raman spectroscopy” and “machine learning” form the central hub, surrounded by clusters associated with algorithmic approaches (deep learning, SVM, chemometrics, PLS-DA), application domains (biomedical diagnostics, microplastics, food analysis), and complementary modalities (SERS, hyperspectral imaging). The overlay visualization further highlights temporal trends, indicating a recent shift toward deep-learning-based models, explainable AI, and interdisciplinary applications. This bibliometric analysis was not intended to provide an exhaustive survey of all published studies [15]. Instead, it was used as a guiding framework to identify representative research directions and to inform the structured selection of case studies discussed in subsequent sections. Similar bibliometric-driven strategies have been successfully used to rationalize literature selection and thematic organization in recent spectroscopy and environmental-analysis reviews [16].

## 3. ML Techniques for Raman Spectral Classification Tasks

### 3.1. Traditional ML Models

Traditional ML algorithms remain widely used in Raman-spectral classification, with representative methods including SVM [17,18], k-nearest neighbors (KNN) [19], decision trees (DT) [20] and RF [21]. These classifiers are often paired with feature-extraction and dimensionality-reduction techniques—such as principal component analysis (PCA) [22], linear discriminant analysis (LDA) [23] and partial least squares discriminant analysis (PLS-DA) [24]—to enhance performance. In many cases, PCA, LDA and PLS-DA not only reduce dimensionality and extract salient features for downstream classification but may also serve directly as discriminant models in specific tasks [25,26].

KNN is a nonparametric, distance-based method that requires no explicit training and is straightforward to implement; however, its accuracy heavily depends on the choice of distance metric and the number of neighbors [27]. SVM constructs an optimal hyperplane and leverages kernel functions to perform nonlinear classification in high-dimensional spaces, offering strong generalization at the cost of more complex parameter tuning [28]. DTs classify via a tree-structured series of decision rules, providing clear interpretability but being prone to overfitting [29]. RF mitigates this risk by aggregating multiple DT, thus improving accuracy though incurring higher computational cost [30].

Overall, traditional ML models offer rapid training and inference, simple architectures, and strong interpretability—qualities that make them particularly suitable for Raman-spectral classification scenarios with limited sample sizes, stringent interpretability requirements or demands for rapid deployment. Owing to their mature algorithmic frameworks and low computational overhead, these methods continue to occupy a central role in Raman-spectral data analysis.

### 3.2. DL Models

In contrast to traditional ML, DL constructs multi-layer neural network architectures that automatically learn complex, nonlinear features directly from raw or minimally preprocessed Raman spectra, thus obviating manual feature-engineering steps. Deep models excel at capturing subtle shifts in peak positions, intensity variations and other fine-grained spectral signatures, yielding outstanding performance on high-dimensional, complex datasets. Widely adopted architectures include artificial neural networks (ANNs) [31], convolutional neural networks (CNNs) [32], recurrent neural networks (RNNs) [33] and generative adversarial networks (GANs) [34].

CNNs leverage convolutional and pooling layers to extract local spectral patterns, proving highly effective in spectrum-pattern recognition [35]; RNNs, with their sequential processing capabilities, adeptly model the ordering of Raman shifts [36]; GANs provide a powerful data-augmentation strategy by generating synthetic spectra to bolster training sets and improve learning under limited-sample conditions [34]. Furthermore, transfer learning has emerged as a practical route to mitigate overfitting when data are scarce, by fine-tuning pretrained models on target Raman-classification tasks to enhance generalization.

Although DL demands substantial data and computational resources, trained models offer rapid inference, making them well suited for real-time detection scenarios. Thanks to their autonomous feature extraction capabilities and streamlined analysis pipelines, DL approaches have become one of the directions for intelligent Raman-spectral classification. Table 1 summarizes representative ML and DL approaches for Raman spectral classification across their quantitative performance and practical strengths. These Raman-specific characteristics influence model selection. For small or moderately sized labeled datasets, latent-variable embeddings (PCA/PLS) combined with linear or kernel classifiers (LDA/SVM) often provide stable performance and clearer interpretation. When spectra are acquired under low laser power or short integration times and become noisy, 1D-CNN/ResNet-type models can better learn local spectral patterns and improve robustness. For deployment across laboratories or instruments, performance should be assessed using grouped or leave-one-group-out validation to avoid optimistic estimates, and the harmonization of calibration/preprocessing protocols becomes critical.

### 3.3. Representative ML Frameworks and Toolkits for Raman Spectral Analysis

In addition to general-purpose ML models, several Raman-specific frameworks and algorithms have been developed to address spectral structure, interpretability, and workflow standardization. These include deep-learning architectures optimized for stimulated Raman scattering or spectral denoising (SRS-Net, SSNet), physically informed or interpretable models such as peak-sensitive logistic regression (PSE-LR), and open-source software toolkits such as PyFasma and RamanSPy that integrate preprocessing, feature extraction, and classification within reproducible pipelines [45,46]. Such task-oriented methods complement conventional ML and DL models by embedding spectroscopic priors and improving transparency and usability.

## 4. Applications of ML in Raman-Spectral Classification

### 4.1. Previous Reviews

The field of Raman spectroscopy, when combined with ML, is advancing rapidly, and several recent reviews have been published to summarize this progress. These reviews offer valuable insights into specific applications and highlight the common challenges and future opportunities within this interdisciplinary area. Several researchers have focused their reviews on different aspects of this technology. For instance, Boateng [47] reviewed the use of DL for Raman spectral analysis, including preprocessing, classification, and regression. This review highlighted the benefits and drawbacks of DL compared to traditional methods and outlined pathways for creating models that are more efficient, interpretable, and generalizable. Similarly, Tang et al. [48] examined key developments in using DL alongside Raman spectroscopy, with a focus on areas like spectral preprocessing, identifying chemical components, and diagnosing diseases. Their work pointed out challenges such as limited data, issues with model generalization, and the need for better interpretability. Chen et al. [49] reviewed how surface-enhanced Raman scattering (SERS) combined with ML is being used for the accurate diagnosis of cervical cancer. Qi et al. [50] offered a broader summary of ML-assisted Raman spectroscopy research across material identification, biological detection, and environmental monitoring, discussing challenges and future directions in data processing, model selection, and practical deployment. Huang et al. [51] provided a comprehensive review of Raman spectroscopy for assessing the quality of fruits, with a particular emphasis on early disease detection, analyzing pesticide residues, and tracing the geographical origin of produce. These reviews demonstrate that researchers are actively synthesizing and evaluating the latest progress, confirming that the integration of ML and Raman spectroscopy is a critical area of scientific inquiry. Table 2 summarizes representative studies (domain, model, dataset size, validation, and accuracy) to facilitate cross-study comparison.

### 4.2. Biomedical Applications

One of the most impactful uses of Raman spectroscopy combined with ML lies in biomedicine [63]. The label-free capability of Raman spectroscopy to probe biochemical constituents in tissues and biofluids has made it invaluable for clinical diagnostics [64]. ML models can efficiently extract subtle spectral differences from complex datasets and translate them into clinically meaningful insights, enabling tasks such as distinguishing diseased from healthy tissue, identifying pathogens and monitoring metabolic changes.

#### 4.2.1. Oncology Applications

**Figure 2:**
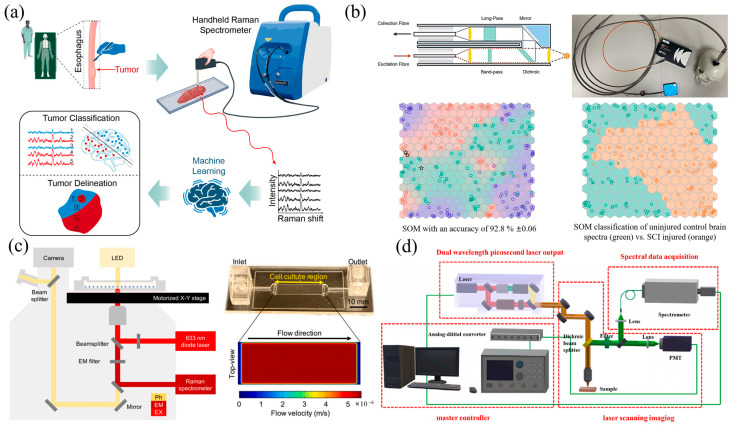
Representative Raman-based techniques for biomedical and clinical diagnostics. (**a**) Intraoperative tumor diagnosis and margin delineation using handheld Raman spectroscopy [53]. (**b**) Intracranial probe integrated with SKiNET for real-time brain injury classification and monitoring [65]. (**c**) Live cell detection via integrated microfluidic chip, phase-contrast imaging, and Raman spectroscopy [42]. (**d**) Custom-built CARS system for highly sensitive imaging of cervical cancer [73].

Raman spectroscopy is frequently employed for real-time tumor detection. Sciortino et al. [52] applied extreme gradient boosting (XGBoost) and SVM to discriminate glioma subtypes and genetic mutations from over 2000 patient biopsy spectra, successfully isolating mutation-associated spectral features. Yang et al. [53] combined a portable Raman spectrometer with ML to collect 1482 spectra from 33 patients, achieving a rapid esophageal tumor-diagnosis model with 92.9% binary classification accuracy—demonstrating the potential of handheld devices and ML for intraoperative diagnosis and margin assessment. As shown in Figure 2a, their workflow integrates handheld Raman scanning of resected tissues with ML-based classification for real-time tumor boundary delineation. Daniel et al. [54] employed Raman spectra with chemometric workflows (PCA followed by KNN or SVM) to characterize pediatric tumor patient blood cells; their SVM model achieved 97.7% accuracy under five-fold cross-validation, supporting rapid blood-based screening. Oncology-oriented Raman + ML studies fall into two practical deployment modes: (i) intraoperative/bedside decision support with portable devices and rapid inference, and (ii) minimally invasive screening using biofluids or blood-derived components. Reported high accuracies should be interpreted together with cohort diversity and validation design, as these factors typically dominate real-world generalizability.

#### 4.2.2. Neurological Applications

For early detection of neurological disorders, Raman spectroscopy coupled with ML has shown excellent performance. Ryzhikova et al. [44] developed a saliva-based Raman assay using genetic-algorithm feature selection and an artificial neural network (ANN) classifier to distinguish Alzheimer’s disease (AD) patients, individuals with mild cognitive impairment (MCI) and healthy controls with 99% accuracy. Stickland et al. [65] designed an optimized intra-cranial Raman-spectroscopy probe paired with the SKiNET ML algorithm for the immediate classification and monitoring of traumatic brain injury, achieving 94.5% accuracy. As shown in Figure 2b, the system integrates a coaxial optical structure with a portable setup and enables accurate spectral classification of both transcranial and SCI-induced brain injuries using self-organizing maps. Moreover, Sarathkumar et al. [66] introduced a surface-enhanced Raman scattering–based lateral flow assay integrated with ML for blood-based neurological biomarker detection, targeting neurofilament light chain as an indicator of neuroaxonal injury and Alzheimer’s disease progression. By combining plasmonic nanohybrids with principal component analysis and a multilayer perceptron classifier, their approach enabled group-wise discrimination among AD, MCI, and control cohorts, demonstrating the feasibility of translating Raman/SERS–ML pipelines into portable, point-of-care diagnostic formats. These neurological applications illustrate a clear translational trajectory, progressing from benchtop Raman spectroscopy toward probe-based, bedside, and point-of-care acquisition with near-real-time ML-assisted classification. In such clinical settings, robustness against acquisition variability—including probe geometry, tissue heterogeneity, and biofluid matrix effects—becomes as critical as classifier performance itself.

#### 4.2.3. Pathogen Identification

Pathogen detection represents a critical application of Raman spectroscopy combined with ML, offering minute-scale diagnostics compared to traditional culture methods. Ho et al. [67] trained a deep convolutional neural network on Raman spectra from 30 microbial species, achieving approximately 99.7% identification accuracy. The COVID-19 pandemic spurred further advances: Chen et al. [68] constructed a stacked ensemble classifier using patient serum spectra, reaching 98% accuracy in infection status prediction; Ember et al. [69] demonstrated noninvasive COVID-19 screening via saliva spectra and ML. Multiple Raman/SERS + ML works have demonstrated COVID-19 detection from saliva or serum samples [70,71], including Raman-based saliva fingerprinting and SERS-assisted ML pipelines, highlighting the rapid translation of spectroscopy + AI workflows toward point-of-care screening during the pandemic. A recent comparative case study evaluated a traditional ML model (PLS-DA) versus a DL model (1D-CNN) for pathogenic microbe identification using an open dataset of 12,000 Raman spectra [72]. The study showed that 1D-CNN achieved higher accuracy/AUC when the full dataset was available, whereas PLS-DA could outperform the DL model when the retained spectra number was reduced, indicating a clear data-size dependence of “ML vs. DL” performance. Importantly, both approaches supported interpretation (PLS-DA loadings, CNN saliency maps) and highlighted similar biochemical bands related to DNA/proteins. For pathogen identification, model choice should be guided by available spectral volume and validation realism, rather than assuming DL is universally superior. Comparative benchmarking and interpretable attribution can help connect classification decisions to biologically meaningful Raman bands.

#### 4.2.4. Cancer Screening

Cancer screening has also emerged as a prominent focus. Lai et al. [42] developed an automated platform combining phase-contrast microscopy with CNNs (U-Net++ and VGG-16) for single-cell metabolic activity classification, with the VGG-16 model achieving 89% accuracy. As depicted in Figure 2c, their system integrates microfluidic trapping, optical imaging, and Raman detection to enable live cell analysis. Liu et al. [73] integrated spontaneous Raman with coherent anti-Stokes Raman scattering (CARS) imaging and employed a ConvNeXt architecture to classify cervical cancer tissues, yielding 100% accuracy. Their dual-modal setup, shown in Figure 2d, combines high-sensitivity imaging with DL for cancer tissue classification. Yang et al. [74] used a multi-model ensemble to subtype breast cancer via Raman spectra, achieving an overall accuracy of 96.77%, thereby revealing subtype-specific biochemical signatures and underscoring the potential for precision diagnosis and personalized therapy. Screening-oriented workflows increasingly emphasize throughput and multimodal integration (Raman with imaging or microfluidic handling). Here, the practical value often lies in standardized pipelines that can output actionable labels at cell/tissue scale while remaining compatible with clinic-friendly acquisition times.

#### 4.2.5. Fundamental Biological Studies

Beyond clinical applications, Raman-ML approaches have been widely adopted in basic biological research. Omucheni et al. [37] proposed a rapid mosquito vector species identification method using Raman spectra and an SVM classifier, achieving 99.7% accuracy after preprocessing. Zhang et al. [38] combined image processing with K-means clustering to classify freshwater mussel inner-shell colors, and, through Raman and microstructural analysis, elucidated differences in organic pigment content and shell layering. Aghasanli et al. [75] applied DL with transfer-learning techniques to migrate models trained on inorganic mineral spectra to organic ivory samples, obtaining up to 99.7% accuracy; notably, the approach maintained 92% accuracy even with unlabeled data, addressing sample scarcity and improving interpretability. Fundamental studies demonstrate that Raman + ML can serve as a scalable phenotyping tool across species/material categories, and transfer learning or weakly labeled strategies can reduce dependence on large, domain-specific labeled datasets.

In summary, the fusion of Raman spectroscopy with ML has transitioned from laboratory proof-of-concepts to promising clinical and biological tools. ML algorithms adeptly handle complex, variable bio-spectral datasets, enabling precise disease discrimination, real-time surgical guidance and rapid pathogen screening. Beyond classification accuracy, recent studies have emphasized model interpretability in biomedical Raman analysis. Ye et al. [76] demonstrated that gradient-based explanation methods can identify physically meaningful Raman bands associated with viral biomolecules, enabling direct linkage between deep-learning decisions and underlying biochemical signatures.

### 4.3. Applications in the Food Sector

Food safety and quality assessment represent another vital arena for the integration of ML with Raman spectroscopy. Given the complex composition of food matrices and their vulnerability to adulteration and contamination, Raman spectroscopy offers rapid, non-destructive “fingerprint” analyses for authenticity verification, while ML enables efficient interpretation of spectral data to detect anomalies and classify product types [55,56].

Food adulteration—driven by economic incentives to substitute premium ingredients with cheaper alternatives—poses significant health risks to consumers. Chen et al. [77] employed Raman spectroscopy combined with a bespoke machine-learning workflow (multiplicative scatter correction preprocessing, genetic-algorithm feature selection, k-means clustering and cubist regression trees) to successfully distinguish Atlantic salmon from low-cost rainbow trout adulteration. Robert et al. [57] integrated Raman spectra with PLS-DA and SVM algorithms to achieve rapid classification of red meats in just 15 s per sample, demonstrating the potential of handheld Raman devices for on-site authenticity testing. Vafakhah et al. [78] compared multiple chemometric approaches (PCA-LDA, CART, SIMCA, PLS-DA and soft-independent modeling of class analogy [SIMCA]) for rice quality control and adulteration detection, finding that MSC preprocessing paired with a soft-kernel machine (SKM) classifier on the 200–1600 cm^−1^ region achieved 98.3% classification accuracy and 100% adulteration-detection precision—outperforming FT-IR data and other models. The effectiveness of different ML workflows across these food-authentication tasks underscores the need for tailored solutions. The optimal choice of a classification model and validation strategy—for instance, whether to validate by sample or by production batch—is highly dependent on the specific food matrix and the commercial context, indicating there is no one-size-fits-all approach.

In the dairy and edible oil industries, Raman–ML approaches have likewise shown excellent performance. Zhao et al. [79] utilized Raman spectral analysis of fatty-acid profiles alongside PCA and RF models to accurately classify different edible oil types. Nunes et al. [40] applied Fourier-transform Raman (FT-Raman) spectroscopy in conjunction with PLS-DA to detect multi-walled carbon nanotubes (MWCNTs) in raw milk with high sensitivity (limit of detection: 0.1 µg/mL) and achieved 100% and 90% identification efficiencies on the training and test sets, respectively. Honey adulteration has also received considerable attention: Oroian et al. [80] combined Raman spectroscopy with multiple machine-learning classifiers (SVM, probabilistic neural network [PNN] and CNN) to discriminate pure honey from adulterated samples; the PNN model achieved a classification accuracy of 96.5%, markedly improving detection efficiency.

**Figure 3:**
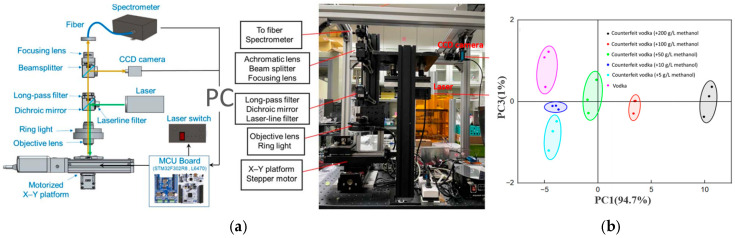
(**a**) Automated Raman spectroscopy system for counterfeit liquor detection and (**b**) PCA-based classification results [81].

Chi et al. [81] developed an automated Raman-spectroscopy platform combined with PCA for both authenticity verification and brand discrimination of alcoholic beverages. By quantifying the Raman-peak intensities of ethanol and methanol, the system enabled precise determination of alcohol content and detection of both high-level and trace methanol adulteration, while successfully differentiating among commercial spirit brands—demonstrating the promise of automated Raman platforms for food-safety surveillance. As shown in Figure 3, the authors integrated a custom-designed motorized X–Y platform with optical components and software control (left), achieving automated spectral acquisition. The corresponding PCA plot (right) reveals clear spectral discrimination between authentic and methanol-adulterated vodkas at varying concentrations, highlighting the system’s capability for both qualitative classification and quantitative analysis. Narvaez et al. [82] proposed a noninvasive, label-free approach for real-time visualization of temperature distributions in cooked pork, employing Raman spectroscopy together with a PCA–RF pipeline. This method achieved 87.5% classification accuracy, offering a novel solution for monitoring thermal processing without damaging samples. In summary, the fusion of Raman spectroscopy with ML has proven highly effective for detecting food adulteration, ensuring product quality and enhancing safety monitoring. Its non-destructive, real-time and high-accuracy characteristics position it as a powerful analytical technology in the field of food science.

### 4.4. Mineral Classification

**Figure 4:**
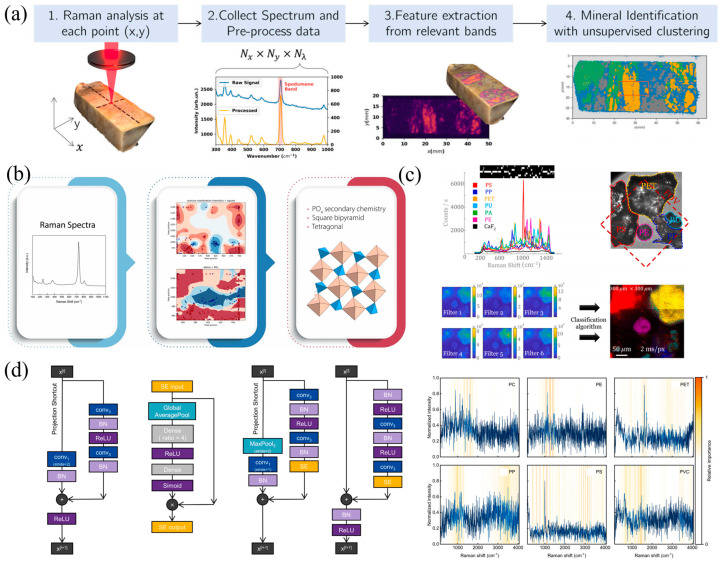
Representative Raman spectroscopy pipelines and DL strategies for unsupervised classification, material identification, and spectral interpretability. (**a**) Unsupervised mineral classification via spatial–spectral Raman imaging [58]. (**b**) Smart Spectral Matching framework for chemical structure recognition [84]. (**c**) Filter-based Raman analysis and RGB mapping for microplastic detection [88]. (**d**) Improved SE-ResNet architecture and Grad-CAM–based interpretation of polymer-specific Raman features [41].

In mineralogy and geological studies, the combination of Raman spectroscopy and ML has demonstrated exceptional capabilities for automated identification and classification. The inherent complexity and diversity of mineral spectra challenge traditional manual methods, whereas machine-learning approaches substantially improve both efficiency and accuracy. Sang et al. [83] further developed a deep convolutional neural network (CNN) model trained on the RRUFF database, attaining 98.4% accuracy and millisecond-scale inference—features ideally suited for in-field and planetary exploration applications. Guimarães et al. [58] developed an unsupervised scheme that extracts diagnostic spectral bands for K-means clustering of Raman images, enabling automatic differentiation of spodumene and petalite without labeled data. The use of radar-plot visualization enhances interpretability, showcasing rapid, cost-effective applications in lithium exploration. As illustrated in Figure 4a, their framework integrates spatially resolved Raman scanning, spectral preprocessing, and unsupervised clustering to generate mineral distribution maps. Smith et al. [84] designed an interpretable classification system that integrates Raman spectra with crystallographic data from the CURIES database to accurately identify secondary anion chemistry and physical structure in uranium-bearing minerals. By correlating key spectral regions with crystal-structure features, this approach outperforms conventional spectral-matching techniques for unknown mineral discrimination. Figure 4b outlines the Smart Spectral Matching pipeline, linking spectral features with structural motifs for accurate phase recognition. Dai et al. [85] proposed an integrated spectroscopic platform combining laser-induced breakdown spectroscopy (LIBS) with Raman spectroscopy (RS) for rapid mineral classification. Their pipeline—comprising data preprocessing, t-SNE visualization, Fisher-score feature selection, and modeling via PLS-DA and kernel extreme learning machine (K-ELM)—yielded 98.4% accuracy on fused LIBS–RS data, offering a powerful tool for geoscientific and industrial mineral surveying.

In archaeometric contexts, Díez-Pastor et al. [59] combined logistic regression, SVM, LDA, DT and ridge regression to trace the provenance of serpentinite minerals. Their SVM model achieved approximately 98% accuracy in cross-validation, providing cultural-heritage researchers with a precise, high-throughput sourcing tool. For planetary science, Johnsen et al. [86] engineered a dual-wavelength (532 nm and 785 nm) Raman spectrometer paired with a multimodal neural network (MNN) to autonomously classify rock-forming minerals on extraterrestrial surfaces. Testing on 191 multi-mineral terrestrial rock samples, their MNN achieved 91% accuracy for pure minerals and 73% for mixed assemblages—underscoring the robustness and real-time decision-making potential of dual-band Raman in lunar and Martian missions. In conclusion, integrating ML with Raman spectroscopy substantially improves mineral identification accuracy and throughput, supporting applications in archeology, planetary exploration, and resource prospecting. Comparing these methodologies highlights a key strategic choice in mineralogical analysis: the distinction between supervised and unsupervised approaches. While supervised models are optimized to achieve the highest possible accuracy for a known classification problem, unsupervised methods excel in exploratory contexts, enabling mineral differentiation and mapping without pre-existing labeled data, thereby prioritizing discovery and interpretability.

### 4.5. Applications in Plastic Materials Detection

The recycling of polymer and plastic materials, as well as monitoring environmental plastic pollution, represents another key application area for the integration of Raman spectroscopy with ML. Given the growing global concern over plastic contamination, rapid and accurate identification of plastic types is crucial for advancing the circular economy and reducing environmental burden. In the context of plastic sorting and recycling, Musu et al. [87] combined PCA with SVM to achieve high-precision classification of polyethylene, polypropylene and polyethylene terephthalate, attaining accuracies above 95%. Furthermore, well-trained ANNs can approach 100% classification accuracy, effectively meeting the real-time identification demands of recycling facilities.

Addressing the challenges of microplastic detection in environmental samples, Fang et al. [60] used Raman micro-imaging coupled with PCA to extract key spectral features without reliance on reference libraries. Their ML pipeline enabled accurate identification and quantification of microplastics in water and soil samples, demonstrating the technique’s versatility from nanoscale to macroscopic monitoring. Grand et al. [88] introduced a high-speed microplastic classification approach based on compressed Raman spectroscopy, employing binary spectral filtering and single-pixel detectors to achieve high-resolution imaging and classification of six microplastic types in marine environments—offering an efficient solution for environmental surveillance. As shown in Figure 4c, the method uses reference-based filter design and projection mapping to enable rapid visual classification of microplastic species at the microscale. Rapid identification of plastics used in beverage packaging has similarly benefited from these methods. Liu et al. [61] compiled Raman spectra for 13 common bottle and cap materials, constructed a representative spectral database and applied t-SNE and PCA for visualization and dimensionality reduction. Among seven ML classifiers evaluated, the RF model performed best, achieving 100% accuracy for bottle-body materials and over 95% for caps, underscoring its practical potential in beverage-bottle recycling. Huang et al. [41] proposed an enhanced residual neural network (SE + Improved ResNet18) for classifying low-quality Raman spectra of microplastics acquired under non-ideal experimental conditions—low laser power and short integration times. By integrating a Squeeze-and-Excitation module and optimizing the residual blocks, their model achieves significantly improved recognition accuracy on high-noise spectra—up to 97.83%—without substantially increasing parameter count or computational overhead. Moreover, they employed Gradient-weighted Class Activation Mapping (Grad-CAM) to visualize the spectral regions driving the network’s decisions, thereby enhancing interpretability. Figure 4d presents the network architecture and Grad-CAM heatmaps highlighting polymer-specific Raman bands under adverse measurement conditions. Benchmarking these different approaches reveals the critical importance of dataset scale and validation rigor. A model demonstrating perfect accuracy on a limited dataset with a simple train/test split may not be as reliable as a model that achieves slightly lower, yet still high, accuracy on a much larger and more challenging dataset using robust cross-validation. The latter provides a stronger indicator of the model’s ability to generalize new, unseen data under real-world conditions.

In the more demanding arena of nanoplastic detection, Gong et al. [89] developed a microfluidic platform using a 1% agarose micro-filter chip and micro-pillar arrays to concentrate polystyrene nanoplastics (PSNPs) from water. After drying, the enriched PSNPs form a transparent film that markedly enhances the Raman signal. Coupled with a CNN for spectral analysis, this system significantly accelerated Raman mapping and achieved precise detection at concentrations as low as 6.25 µg/mL, demonstrating its promise for rapid, high-sensitivity monitoring of nanoplastic pollution in complex environments.

Despite these advances, the practical deployment of ML-assisted Raman systems for microplastic detection still faces some challenges. Miniaturization and real-time analysis using handheld Raman devices often require compromises in laser power and acquisition time, leading to lower signal-to-noise ratios and degraded classification performance. Environmental factors such as moisture, surface contamination, and fluorescence further complicate in situ measurements. Recent studies have shown that robust ML models must therefore be trained on spectra acquired under variable experimental conditions, rather than ideal laboratory settings, to ensure reliability in field applications [90]. In addition, the integration of Raman spectroscopy with imaging modalities—such as Raman mapping or hyperspectral imaging—offers valuable spatial information on microplastic distribution, but also generates large, high-dimensional datasets that demand efficient data reduction and fast inference strategies for real-time use.

### 4.6. Applications in Other Domains

The fusion of ML with Raman spectroscopy has unlocked unique possibilities across forensics, art conservation, archeology and pesticide detection, greatly extending the analytical reach of Raman methods. In forensic science, the rapid and accurate identification of trace evidence at crime scenes is critical. Raman’s non-destructive nature makes it ideal for analyzing minute samples. Liu et al. [62] applied PCA for feature extraction on Raman spectra from disposable face masks and then compared classifiers—including SVM, Bayesian discriminant analysis and back-propagation neural networks—to determine manufacturing origin. The Bayesian model achieved 100% accuracy, demonstrating that Raman spectra can capture manufacturer-specific signatures and provide a powerful tool for provenance determination. Koçak et al. [39] combined attenuated total reflectance infrared and Raman spectra, using RF, SVM and k-NN to classify nail-polish samples. The RF classifier on the fused dataset reached 99.95% accuracy, and feature-importance analysis identified key functional groups, yielding a highly automated, interpretable framework for forensic evidence identification. From a forensic validation standpoint, it is crucial to consider the scale and methodology of the studies. Findings derived from larger datasets that are tested with more rigorous validation techniques inherently provide greater confidence in the model’s reliability and robustness compared to results from smaller-scale studies with more limited hold-out testing.

In art conservation, Raman’s ability to analyze pigments and substrates without sampling has made it invaluable for cultural-heritage studies. Yan et al. [91] investigated Raman spectra of 18 handmade papers from antique books and artworks, comparing five machine-learning models; a PCA-logistic-regression approach delivered the highest classification and prediction accuracy, confirming the method’s ability to distinguish paper types precisely. Qi et al. [92] developed a CNN-based method to automatically identify common white mineral pigments in artworks, achieving 98.7% accuracy. This deep-learning approach markedly outperformed traditional techniques, providing a powerful, noninvasive tool for authentication and pigment provenance studies.

In pesticide detection, Yüce et al. [93] built a custom 785 nm–excitation Raman spectrometer and compiled a fingerprint database for 14 common pesticides. Using multivariate analyses (PCA, hierarchical cluster analysis) alongside an RF classifier, they achieved efficient pesticide discrimination. Comparison with a commercial 532 nm system highlighted the superior fluorescence suppression afforded by the 785 nm excitation, underscoring Raman’s promise for food-safety monitoring. Sakrabani et al. [94] pioneered the integration of neutron computed tomography (NCT), X-ray computed tomography (XCT) and Raman spectroscopy to characterize organic-inorganic fertilizer granules. While NCT and XCT provided complementary data on particle density and porosity, RS revealed inorganic composition despite fluorescence interference from organic binders, demonstrating the feasibility of this multimodal strategy for fertilizer quality control.

## 5. Challenges and Future Directions

### 5.1. Current Challenges

Despite notable progress in coupling ML with Raman spectroscopy across diverse applications, several critical challenges remain. A primary issue lies at the data level, where sample collection and ethical constraints are particularly acute in biomedical studies. Obtaining patient tissue samples typically requires stringent ethical approvals and informed-consent procedures, which limit both the size and diversity of available datasets. To address this, Esteves et al. [95] proposed using low-ethics-burden alternatives like the chorioallantoic membrane model in chicken embryos for early-stage validation. Stemming from data scarcity, the generalization of DL models on Raman data remains problematic. Sample preparation and spectral acquisition are time- and labor-intensive, making it difficult to assemble large, well-annotated datasets. Although data-augmentation techniques, such as noise injection and GANs, can partially bridge this gap, they often fail to capture the full spectrum of natural sample variability [96]. Moreover, systematic differences between laboratories and instrumentation further hinder data integration and model generalization. Validation studies have shown that these issues are not merely theoretical but manifest clearly in practice. Lilek et al. [97] compared cross-validation strategies on biological Raman/SERS datasets. They showed that unstratified K-fold and leave-one-out can overestimate performance when spectra from the same replicate are not properly grouped. More realistic designs (leave-one-group-out) yielded lower but more reliable estimates of generalization. In contrast, more realistic strategies such as leave-one-group-out validation consistently yielded lower but more representative accuracies, highlighting the risk of overfitting and poor cross-laboratory generalization in many existing studies. The establishment of large-scale, standardized shared databases and consensus protocols for instrument calibration and data formatting is essential to enhance both data interoperability and model robustness.

To facilitate cross-institutional data sharing and improve model generalization, recent open-science and FAIR-oriented studies have emphasized the need to mandatorily report a minimal yet standardized set of Raman metadata. Key metadata points include: (i) instrument information (manufacturer, model, laser wavelength, spectral resolution); (ii) acquisition parameters (laser power at sample, integration time, number of accumulations, objective and numerical aperture); (iii) calibration and preprocessing details (wavenumber calibration standard, intensity calibration, baseline correction and normalization methods with parameters); (iv) sample-related metadata (sample type, preparation protocol, substrate, environmental conditions); and (v) data provenance information (file format, software versions, preprocessing workflow, and licensing) [98]. The consistent reporting of these metadata elements is increasingly recognized as essential for reproducibility, database interoperability, and reliable cross-laboratory model validation [99].

Another underappreciated challenge is the use of Raman band intensities for quantitative or semi-quantitative inference. In practice, peak intensities are affected not only by the amount of a chemical group but also by molecular conformation and measurement conditions, meaning that intensity-based ratios or peak areas are not uniquely determined by concentration. This limitation is closely tied to Raman–ML classification, because class labels are often defined by such semi-quantitative criteria (such as composition grades, adulteration level, or biochemical content), and models may inadvertently learn instrument-specific intensity signatures rather than chemistry, harming generalization. Samyn et al. [100] combined FT-Raman spectra with multivariate modeling to estimate oil quality indices, illustrating that reliable “quantification-like” outcomes depend on validation design. Taieb et al. [101] demonstrated that handling of Raman intensities within a clinically oriented classification pipeline (guided by quantitative phase imaging) can influence diagnostic decision boundaries. Therefore, Raman–ML studies should explicitly distinguish fingerprint-based classification from quantitative claims, and treat mechanistic interpretation based on band-intensity changes as conditional on acquisition control and validated calibration.

Directly related to these challenges is the issue of reproducibility. The rigor of many published findings is difficult to assess independently because the public availability of raw spectral data and the code used for preprocessing and model training is the exception rather than the rule. Without access to these resources, it is nearly impossible to replicate reported results or test models on new data. Additionally, subtle variations in sample preparation and instrument parameters are often under-reported but can drastically affect model performance. Therefore, even with high reported accuracies, the lack of standardized protocols and open data practices presents a significant barrier to validating the robustness and generalizability of these AI-driven Raman classification systems. Finally, the computational cost and real-time deployment of complex models are significant issues. DL models have high computational demands, making them difficult to deploy on portable or embedded devices. The lack of interpretability is another notable issue that limits the application of DL models in sensitive domains. While these models perform exceptionally well, their decision-making mechanisms lack transparency. Developing explainable AI techniques like Grad-CAM is an important direction for improving model transparency [76].

### 5.2. Future Research Prospects

The adoption of explainable artificial intelligence (XAI) in Raman spectroscopy has shown clear domain-specific trends in recent years [102,103]. In biomedical applications, XAI techniques such as Grad-CAM, SHAP, and masking-based methods are increasingly used to link model decisions to biologically meaningful spectral regions, supporting clinical interpretability and trust. In food and agricultural analysis, model-agnostic approaches—particularly SHAP and LIME—are favored to identify chemically relevant bands associated with composition, quality, or adulteration. In contrast, mineralogy and materials science have adopted global or physics-informed explanation strategies, combining dimensionality-reduction methods with sensitivity analysis to relate spectral variability to structural or compositional factors, often within multimodal characterization frameworks.

To address the challenges in data scarcity, model generalization, research reproducibility, and practical deployment, future research must pursue several key directions. First, efforts must expand the application of ML-assisted Raman spectroscopy into underexplored domains—such as corrosion-product classification, environmental-pollution monitoring and analyses of complex biological systems—by developing bespoke, open-architecture deep-learning models optimized for Raman data and real-time, high-efficiency inference. Second, multimodal integration should be advanced by combining Raman spectroscopy with complementary platforms—such as microfluidics, mass spectrometry and other cutting-edge analytical techniques—to create comprehensive measurement suites. Such platforms promise breakthroughs in profiling bacterial phenotypes, elucidating antibiotic-resistance mechanisms and monitoring single-cell dynamics. Third, the latest generative AI approaches should be harnessed to enrich spectral-data augmentation and interpretation, accelerating the discovery and characterization of novel spectroscopic biomarkers for microbiology, drug development and clinical diagnostics. Finally, the field must establish clear standard operating procedures for sample preparation, spectral acquisition and data analysis, along with community-endorsed guidelines to promote cross-institutional and cross-disciplinary data sharing. By driving efforts in data standardization, model refinement, interdisciplinary collaboration and trust-building through interpretability, the fusion of ML and Raman spectroscopy will realize its full potential—effectively translating laboratory innovations into robust, real-world solutions and serving as a catalyst for breakthroughs across multiple scientific and technological domains.

Beyond advancing feature-based and deep-learning methods, it is equally crucial to develop and integrate holistic spectral analysis strategies. For many applications involving complex systems—such as biological fluids, composite industrial materials, or environmental samples—the significant overlapping of vibrational bands and complex spectral backgrounds can make the unambiguous assignment of peaks to specific chemical components impractical or even impossible [104,105,106]. In such cases, methods that treat the entire spectrum as an indivisible “fingerprint” and quantify its overall similarity or difference to reference spectra are more robust than traditional peak-based approaches.

The spectral distance approach is a prime example of this paradigm. In practice, the effectiveness of holistic, full-spectrum distance metrics in unsupervised or semi-supervised settings relies on embedding spectra into a reduced, physically meaningful subspace prior to distance computation. Dimensionality reduction techniques such as PCA or related latent-variable models concentrate the dominant variance into a limited number of components, enabling distance measures (Mahalanobis or correlation-based metrics) to be evaluated in a low-dimensional and statistically well-conditioned space. This strategy explicitly mitigates the curse of dimensionality while preserving global spectral information, making full-spectrum approaches particularly suitable for high-dimensional Raman data with limited labels. Such pipelines have been successfully applied in biomedical spectroscopy, for example by combining PCA with Mahalanobis distance or LDA to achieve robust classification of cancerous tissues and liquid biopsy samples based on holistic Raman fingerprints [107,108,109,110].

## Figures and Tables

**Table 1:** Comparison of ML and DL Methods for Raman Spectral Classification.

Model Type	Algorithm	Performance Metrics	Key Advantage	Data Type	Ref.
Traditional ML	PCA-QDA	94% (Accuracy)	Small-sample suitability, strong interpretability	Raman spectra	[37]
	K-means clustering	Correct color grouping	Unsupervised, no labeling required	Raman spectra + images	[38]
	RF	99.95% (Accuracy)	Excellent accuracy, strong robustness, high interpretability	ATR-IR + Raman spectra	[39]
	PLS-DA	Near-zero false positives	Chemically interpretable, low detection limit	FT-Raman spectra	[40]
DL	SE-ResNet (CNN variant)	97.83% (Accuracy)	High noise tolerance, suitable for harsh conditions	Raman spectra (low SNR)	[41]
	VGG-16 (CNN)	89% (Accuracy)	End-to-end learning, single-cell resolution	Phase-contrast + Raman	[42]
	Customized ANN	93.8% (Accuracy)	Non-invasive, good generalization	Raman spectra	[43]
	ANN	84% (Sensitivity/Specificity)	Handles subtle biochemical differences	NIR Raman spectra	[44]

**Table 2:** Summary of Representative Studies on ML-based Raman Spectral Classification.

Application Area	Best ML Model	Spectrum Size	Validation Strategy	Accuracy (%)	Year	Ref.
Glioma IDH-mutation classification	RBF-SVM (radial-basis SVM)	2073 spectra from 38 fresh specimens	Leave-one-patient-out (LOPO) + nested 5-fold CV	87	2021	[52]
Esophageal cancer vs. normal tissue detection	SVM	9162 spectra from 40 patients	Train-test split (30 vs. 10 patients) + LOOCV	88.61	2024	[53]
Normal vs. pediatric Leukemia Vs. Non-Leukemic cancer	PLS-DA	308 spectra from 121 blood samples	Cross-validated PLS-DA (8 latent variables)	98.3	2023	[54]
Baijiu (Chinese liquor) authentication	LDA-RF ensemble	480 Raman spectra of Jia Pin	leave-one-bottle-out cross-validation	96.7	2023	[55]
Edible oil authentication and adulteration	Subspace k-NN ensemble	Raman spectra from 36 samples	8-fold cross-validation	88.9	2020	[56]
Beef, venison, lamb discrimination	SVM	The training set contained 60 samples	3-fold cross-validation + independent test set	93–100 (sensitivity/specificity)	2021	[57]
Li-bearing mineral mapping	K-means + interpretable assignment	4 extracted bands (≥1000 spectra)	Independent blind samples	Not quantified	2024	[58]
Variscite mine-of-origin and depth	SVM	100 Raman spectra	5-fold CV	98 (mine)/87–90 (depth)	2020	[59]
Microplastic imaging	PCA-based decoder	7744 spectra	Visual/standard spectrum match	Qualitative	2022	[60]
Plastic beverage-bottle forensic ID	CNN (1-D)	spectral data from a total of 35 samples	7:3 train/test split	100 (training and test)	2025	[61]
Low-quality microplastic spectra	SE-Improved ResNet18	1800 spectra from 6 microplastics	5-fold CV	97.83 (worst-case)	2025	[41]
Forensic identification of disposable masks	Bayes Discriminant Analysis	37 spectra from 37 masks	30-sample training + 7-sample hold-out test	100.0 (both train and test)	2021	[62]

## Data Availability

No new data were created or analyzed in this study. Data sharing is not applicable to this article.
